# Elevated CO_2_ buffers negative impacts of heat stress on short-term growth adjustments of 180-year-old *Quercus robur*

**DOI:** 10.1093/treephys/tpag114

**Published:** 2026-08-20

**Authors:** Anna Gardner, Angus R MacKenzie, Muhammad S Amjad, Stefan Krause, Joshua Larsen, Susan Quick, Klaske van Wijngaarden, Alice Gauthey

**Affiliations:** School of Biological Sciences, University of Birmingham, Edgbaston B15 2TT, UK; Birmingham Institute of Forest Research, University of Birmingham, Edgbaston B15 2TT, UK; Birmingham Institute of Forest Research, University of Birmingham, Edgbaston B15 2TT, UK; School of Geography, Earth and Environmental Sciences, University of Birmingham, Edgbaston B15 2TT, UK; Birmingham Institute of Forest Research, University of Birmingham, Edgbaston B15 2TT, UK; Department of Botany, University of Kotli, Kotli 11100, Azad Jammu and Kashmir, Pakistan; Birmingham Institute of Forest Research, University of Birmingham, Edgbaston B15 2TT, UK; School of Geography, Earth and Environmental Sciences, University of Birmingham, Edgbaston B15 2TT, UK; Birmingham Institute of Forest Research, University of Birmingham, Edgbaston B15 2TT, UK; School of Geography, Earth and Environmental Sciences, University of Birmingham, Edgbaston B15 2TT, UK; Birmingham Institute of Forest Research, University of Birmingham, Edgbaston B15 2TT, UK; School of Geography, Earth and Environmental Sciences, University of Birmingham, Edgbaston B15 2TT, UK; Birmingham Institute of Forest Research, University of Birmingham, Edgbaston B15 2TT, UK; School of Geography, Earth and Environmental Sciences, University of Birmingham, Edgbaston B15 2TT, UK; Hawkesbury Institute for the Environment, Western Sydney University, Penrith, New South Wales 2751, Australia; Birmingham Institute of Forest Research, University of Birmingham, Edgbaston B15 2TT, UK; School of Geography, Earth and Environmental Sciences, University of Birmingham, Edgbaston B15 2TT, UK

**Keywords:** climate change, leaf gas exchange, photosynthesis, tree ecophysiology, water use efficiency

## Abstract

Rising atmospheric carbon dioxide (CO_2_) concentration are a primary driver of global warming and are expected to be associated with more frequent droughts, greater temperatures and increased vapour pressure deficit (*VPD*). These factors are all key drivers of tree mortality. Whilst elevated CO_2_ (eCO_2_) enhances photosynthesis (A_net_) and intrinsic water-use efficiency (_i_WUE), its capacity to mitigate heat stress in mature trees under field conditions remains poorly understood. We investigated the effects of eCO_2_ on the physiological and growth responses of mature *Quercus robur*L. (~180 years old) at a forest Free Air Carbon Enrichment (FACE) experiment. We combined measurements of tree growth, canopy conductance and leaf gas exchange, together with leaf morphological traits, collected during a naturally occurring heat events (>32 °C), to assess whether eCO_2_ buffered the impacts of extreme heat events (>32 °C). The leaf-level measurements also enabled us to determine whether the physiological enhancements previously observed during the early years of Birmingham Institute of Forest Research FACE were maintained following prolonged exposure to CO_2_. After 8 years of CO_2_ enrichment (+150 p.p.m. above ambient), eCO_2_-grown trees showed increased _i_WUE (+33%), driven by increased A_net_ (+26.1%) and modest reductions in stomatal conductance (−11.1%), with no significant changes in stomatal anatomy. Elevated CO_2_ increased the sensitivity of canopy-level conductance to *VPD* during heat events, indicating stronger stomatal regulation under high atmospheric demand in eCO_2_ compared with ambient CO_2_-grown trees. Heat stress reduced tree growth in both conditions, but the reduction was less pronounced under eCO_2_, suggesting partial mitigation of heat stress effects. These findings indicate that eCO_2_ can confer partial physiological buffering against heat stress in mature *Q. robur*, enhancing resilience without compromising structure. Shifts in water use highlight the importance of integrating CO_2_–climate interactions when predicting forest responses to future climate extremes.

## Introduction

Rising atmospheric carbon dioxide (CO_2_) concentrations are driving global warming (e.g., Yoro and Daramola 2020, Calvin et al. 2023), increasing the frequency, duration and severity of climate extremes, including drought and heat stress events (IPCC 2023). These stressors are amongst the primary drivers of tree mortality globally, threatening forest function and stability (Hammond et al. 2022). Despite this, elevated CO_2_ (eCO_2_) concentrations can also enhance photosynthetic carbon uptake (Zhang et al. 2021) and improve intrinsic water-use efficiency (_i_WUE) in trees, potentially mitigating some impacts of warming and drying. Yet, the extent to which eCO_2_ can buffer trees against heat and drought stress remains unclear, particularly in mature forests where long-lived trees have developed complex responses due to their size, age, plant–soil coupling and hydraulic architecture (Körner 2006).

Increases in atmospheric CO_2_ are predicted to alter the physiology and functioning of trees, particularly in relation to water-use processes. For example, eCO_2_ is known to influence tree water use, primarily by increasing _i_WUE (Mathias and Thomas 2021) through reduced stomatal conductance (*g*_s_), often leading to lower transpiration (Hernandez-Santana et al. 2016), and enhanced carbon assimilation (A) (Medlyn et al. 2011, Walker et al. 2021, Wang and Wang 2021). Long-term increases in _i_WUE in response to eCO_2_ have also been observed in tree-ring records (van der Sleen et al. 2015). At larger scales, these responses may alter ecosystem-scale water dynamics through changes in transpiration, root growth patterns and soil moisture dynamics. Notably, eCO_2_-induced enhancement of _i_WUE can improve leaf water status under drought conditions (Wang et al. 2022) and slow soil moisture depletion by reducing whole-tree transpiration, potentially enhancing tree drought resistance (Leuzinger and Körner 2007).

In addition to altering tree water relations, eCO_2_ has been found to increase tree growth (Medlyn et al. 2011, Walker et al. 2021, Wang and Wang 2021), although this has not always been the case (Ellsworth et al. 2017). Growth stimulation under eCO_2_ is typically driven by increased leaf-level photosynthesis and increases in leaf area index (Zhang et al. 2021, Gardner et al. 2022, 2023). However, the magnitude and direction of growth responses vary widely depending on tree species, stand age and environmental conditions (Dawes et al. 2011, Maschler et al. 2022). Short-term elastic changes in stem radius are primarily mediated by water availability and temperature-driven hydraulic limitations. Reduced stomatal conductance (*g*_s_) under eCO_2_ can decrease transpiration whilst maintaining or increasing carbon assimilation, resulting in higher iWUE. Lower transpiration rates may contribute to a more stable plant water balance and slower stem shrinkage during dry periods, potentially supporting continued growth. However, during periods of high temperature and low soil moisture, hydraulic limitations may intensify water potential gradients within the tree, increasing tree water deficit (TWD) (Ziegler et al. 2024). This can lead to temporary reductions in stem radius and negate any short-term benefits of eCO_2_. Although warming and eCO_2_ can both enhance photosynthesis and growth, extreme drought can eliminate these positive effects (Duan et al. 2015).

Heat stress can impose severe physiological impacts on trees, in part by increasing vapour pressure deficit (*VPD*), which increases transpiration rates and accelerates plant water loss (Grossiord et al. 2020). In response to high temperatures and *VPD*, trees typically close their stomata to reduce water loss and preserve the hydraulic integrity of the xylem, thereby reducing the risk of hydraulic failure through embolism formation (McDowell et al. 2008). High temperatures also directly impair photosynthesis by disrupting metabolic proteins and damaging the photosynthetic machinery (Allakhverdiev et al. 2008). In particular, heat can reduce Rubisco activity by degrading Rubisco activase, a heat-sensitive enzyme essential for carbon fixation, thereby increasing photorespiration (Salvucci and Crafts-Brandner 2004, Sage et al. 2008). Importantly, elevated temperatures can impair hydraulic function even in the absence of soil drought, as increased *VPD* alone can induce xylem cavitation (Schönbeck et al. 2022).

Evidence for the capacity of eCO_2_ to mitigate heat and drought stress remains mixed. Whilst some studies report reduced canopy conductance and improved water status under eCO_2_ (Gattmann et al. 2023), these potential benefits may be offset by increases in leaf area or diminish under extreme temperature conditions (Birami et al. 2020). In some cases, stress responses may even be exacerbated under eCO_2_, with greater premature leaf senescence and abscission during hot-dry events in mature stands (Wullschleger and Norby 2001, Warren et al. 2011). Despite these findings, the extent to which eCO₂ buffers against heat-induced damage, particularly in mature, long-established (age >20 years) trees, remains poorly understood. With more frequent, intense and severe heat extremes expected, understanding the influence of eCO_2_ on tree physiological and growth responses is key to predicting forest functioning in a changing climate.

Whilst the individual impacts of eCO_2_, drought and heat stress on tree physiology and growth are relatively well-documented, at least for seedlings in controlled environments, their combined impacts under natural field conditions remain poorly characterized (Umaña et al. 2025). In natural environments, trees are often exposed to multiple stressors simultaneously, and these interactions strongly influence ecohydrological dynamics, tree growth and resilience Zhu et al. 2021). Elevated CO_2_-induced changes in _i_WUE and transpiration may also provide some degree of protection under heat stress. However, reduced transpiration may increase leaf temperature, potentially constraining photosynthetic capacity (Hagan Brown et al. 2025).

In this study, we investigated the influence of eCO_2_ on physiological and growth responses of mature *Quercus robur* (~180 years old) across naturally occurring heat events between 2019 and 2023 under field conditions. Amongst these events, the exceptional 2022 UK heatwave, the warmest year in the instrumental record and one of the most intense August heat events since the late 19th century, provided the strongest test and therefore serves as the principal case study throughout. We combined high frequency measurements of tree growth (i.e., stem radius and woody biomass) with observations of physiological traits (photosynthesis, stomatal conductance and canopy transpiration) to assess whether long-term eCO_2_ exposure (up to 8 years) alters tree sensitivity to heat stress. We hypothesized that trees exposed to eCO_2_ would exhibit greater sensitivity to *VPD* due to higher _i_WUE, thereby maintaining carbon assimilation and growth during heat stress. In contrast, trees under ambient CO_2_ (aCO_2_) were expected to display more conservative stomatal behaviour and reduced final growth following heat stress. By focusing on mature trees under field-based CO_2_ enrichment during an extreme climatic event, this study provides new insight into how long-term CO_2_ exposure interacts with heat stress to influence forest function.

## Materials and methods

### Site

This study was conducted at the Birmingham Institute of Forest Research (BIFoR) Free Air CO_2_ Enrichment (FACE) facility located in Staffordshire (52.801°N, 2.301°W), UK. BIFoR FACE is a ‘second generation’ Forest FACE facility, extending the scope of first-generation facilities (see Norby et al. 2016), and situated within 19 ha of mature northern temperate broadleaf deciduous woodland having a canopy height of 24–26 m. The woodland consists of an overstorey canopy dominated by English oak (*Q. robur* L.) and a dense understorey comprising mostly of hazel coppice (*Corylus avellana* L.), sycamore (*Acer pseudoplatanus* L.) and hawthorn (*Crataegus monogyna* Jacq.). The site was planted with the existing oak standards in the late 1840s and, as is common for many mature temperate forests, has experienced limited active management over the past ~ 40 years.

The study site is located within the maritime zone of the temperate forest climate space characterized by cool, wet winters and warm, relatively dry summers, with a frost-free growing season from April to October (Sommerfeld et al. 2018, Jiang et al. 2020). We focused our study between the years 2019 and 2023. Some of the following measurements were taken outside this range, however, we considered that said measured traits would not change considerably over the years (see Figure S1 available as Supplementary Data at *Tree Physiology* Online). The year 2022 was the warmest in the UK series from 1884 (Kendon et al. 2024), with August 2022 being the second most intense heatwave to have occurred in August since 1878 (Yule et al. 2023).

BIFoR FACE consists of nine approximately circular experimental plots of woodland 30 m in diameter (Hart et al. 2020). Only the six plots with infrastructure were considered in the present study. Each ‘infrastructure plot’ is encircled by bespoke steel towers constructed to reach 2-m above the local canopy-top height. The facility operates with two treatments as three replicate plots: aCO_2_ (ca 405 μmol mol^−1^ during the study period) and eCO_2_. The eCO_2_ plots were supplied with CO_2_-enriched air to achieve a target concentration of +150 μmol mol^−1^ above the ambient CO_2_ concentration (i.e., concentrations in the elevated plots were ca 558 μmol mol^−1^ on average between 2019 and 2023), maintained as 5-min moving averages (Hart et al. 2020). Details of the FACE system’s operation and its statistical performance in achieving target CO_2_ concentrations have been described in Hart et al. 2020; updated in Table S1 of Norby et al. 2024. For this study, the sample size used throughout the study (*n* = 3) represents the number of replicate experimental plots at BIFoR FACE and includes within-tree replicates that were averaged per plot before analysis.

### Climate and hot events

We used weather data from the on-site meterological towers, taking measurements of air temperature and precipitation at top, middle and low canopy levels every hour and continuously, respectively (MacKenzie et al. 2021). Vapour pressure deficit (*VPD*; see, e.g., (Monteith and Unsworth 2013, Flo et al. 2022) was calculated using top-canopy air temperature (*cf.*  Hagan Brown et al. 2025). Our data focused on the top canopy level, where heat stress was expected to be the greatest.

There is currently no standard definition for identifying heat events, and published thresholds vary in duration and intensity (Qiu and Yan 2020). In this study, we focused on prolonged periods of elevated temperature with minimal precipitation events. We defined a ‘hot event’ for each year as the 5-day period in which the sum of daily maximum air temperature (T_air_) at the top of the canopy was the highest. This approach enabled consistent comparisons of tree responses across multiple naturally occurring heat events (2019–23) whilst recognizing that the 2022 heatwave served as the focal case for examining responses under the most extreme conditions experienced during the study period. Corresponding thresholds for air temperature (*T_air_5days_*;  °C) and vapour pressure deficit (*VPD_air_5days_*, kPa), calculated as the average of daily maxima over the 5-day period, are summarized in Table 1. It is worth noting that these periods did not always align with the single highest temperature days of the year (e.g., in 2022, the annual maximum temperature reached 35.9 °C).

**Table 1:** Environmental conditions during hot events and preceding periods. *T_air_5days_* and *VPD_air_5days_* denote the mean of daily maximum air temperature ( °C) and vapour pressure deficit (kPa), respectively, calculated over the 5-day hot event period. *T_air_5days_before_* and *VPD_air_5days_before_* represent the corresponding means over the five consecutive days preceding each hot event (‘priors’).

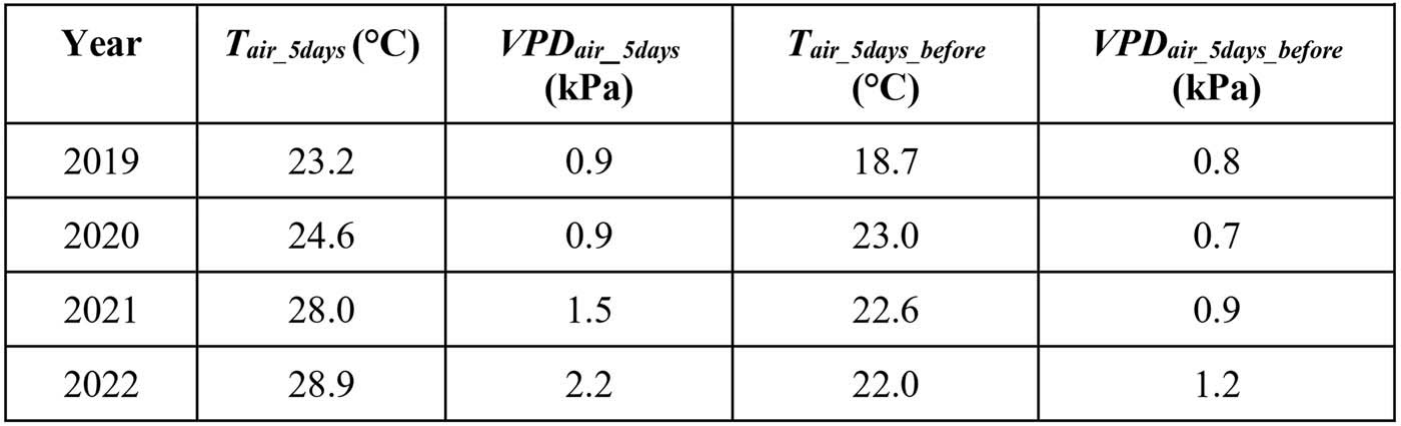

To assess pre-event conditions, we also extracted the five consecutive days prior to each ‘hot events’, hereafter referred to ‘hot event priors’. These periods were characterized by milder environmental conditions whilst maintaining comparable leaf age and biotic disturbance (Table 1, Figure S2 available as Supplementary Data at *Tree Physiology* Online).

### Photosynthetic data

To assess the effects of eCO_2_ alone independent of any individual heatwave event, nine diurnal campaigns of gas exchange were conducted in July and August of 2018, 2019 and 2024 to assess the effect of eCO_2_ on photosynthesis and stomatal conductance in the mature *Q. robur* trees. Each campaign spanned three consecutive days, during which physiological measurements were taken between 09:00 and 18:00 h to capture a range of air temperatures, *VPD* and light conditions. On each day, two FACE plots, one aCO_2_ and one eCO_2_, were measured. Each plot measurement took ~1.5 h, and aCO_2_ and eCO_2_ plots were measured in an alternating sequence within each day to minimize potential bias associated with diurnal variation in environmental conditions (e.g., temperature, light and *VPD*). This approach helped ensure that both treatments were sampled across comparable environmental conditions. In each plot, two trees near the central canopy access tower were selected, and measurements were taken from one sun-exposed leaf per branch, across two branches per tree (*n* = 6 trees per treatment).

Gas exchange measurements (net assimilation, A_net_ and stomatal conductance, g_s_) were conducted on sun-exposed leaves located at the top of the canopy using a LI-COR 6800 portable photosynthesis system (LI-COR Biosciences, Lincoln, NE, USA). Prior to each measurement, leaf chamber temperature and relative humidity were manually adjusted to match the prevailing ambient conditions at the time of measurement in order to maintain stability within the cuvette. Photosynthetically active radiation (PAR) was recorded using the integrated LI-6800 light sensor. Reference CO_2_ concentrations were set to 405–420 p.p.m. for aCO_2_ plots and 555–570 p.p.m. for eCO_2_ plots, reflecting the respective treatment conditions. Measurements were recorded once gas exchange had stabilized, typically within ~5 min of leaf enclosure in the chamber.

The _i_WUE was calculated by dividing A_net_ by g_s_ for each individual (*j*) at time (*t*) as: 


(1) \begin{equation*}{\mathrm{iWUE}}_{j,t}=\frac{{{\mathrm{A}}_{\mathrm{net}}}_{j,t}}{{{\mathrm{g}}_{\mathrm{s}}}_{j,t}}\end{equation*}

### Stomatal traits

To assess whether trees would adjust leaf-level traits related to water regulation under eCO_2_, we measured minimum stomatal conductance (*g*_min_) and stomatal morphology.

From May to August 2024, *g*_min_ was measured monthly. Each month, sun-exposed twigs from the upper canopy were cut from two trees per plot and immediately sealed in moist Ziplock bags to prevent dehydration. Samples were transported to the laboratory at the University of Birmingham campus and processed within 24 h. Measurements followed the protocol of Sack and Scoffoni in Prometheus Protocols (Sack et al. 2010). Briefly, one fully expanded leaf per twig was selected (*n* = 12 per month). Petioles were sealed with parafilm to prevent water loss, and leaf area was measured using a flatbed scanner (model Epson Perfection V850 Pro, Epson, Nagano, Japan). Leaves were then affixed to adhesive tape in a ‘clothesline’ arrangement for a minimum of 1 h to allow stomatal closure. A small fan was placed beneath the setup to accelerate dehydration. Following closure, leaves were weighed at ~ 30-min intervals using an analytical balance (model Entris II, Sartorius Inc., Göttingen, Germany). At each time point, ambient temperature and RH were recorded using a handheld environmental sensor (model M2000C, Temtop, San Jose, CA, USA). After at least eight measurements per leaf, leaf area was re-measured to account for any shrinkage during the drying process. Final *g*_min_ values were calculated using the ‘g_min_ Analysis Spreadsheet Tool’ provided in Prometheus Protocols.

In September 2024, additional leaves were collected using the same method as for *g*_min_ measurements (*n* = 12). For each leaf, surface area was measured using a leaf area metre with belt conveyor (WinDIAS Leaf Area Metre). To assess stomatal traits, a thin layer of transparent nail polish was then applied to the abaxial (lower) surface of each leaf. Once dried, the imprint was carefully peeled and mounted onto a microscope slide (Karl Hecht GmbH, Rhön, Germany). Slides were imaged under a Nikon SMZ1000 stereomicroscope at 20× magnification. Stomatal density (number of stomata per mm^2^) and stomatal length (μm) were quantified using Fiji (ImageJ) software.

### Tree growth data

In April 2019, auto-dendrometers (EMS brno DRL26) were fitted in all infrastructure plots on oak trees with a diameter greater than 10 cm diameter at breast height (DBH) i.e., ~1.3 m height (*n* = 24). Automated circumference change measurements were recorded every 30 min.

Stem growth accumulation for each tree was derived from those high frequency dendrometer measurements using the *treenetprocnet* R package (Knüsel et al. 2021). To account for variation in initial tree size, growth was normalized by calculating a relative growth ratio, defined as growth at time *t* divided by the maximum annual growth for each individual tree.

The TWD (μm) was derived from each tree’s dendrometer data also using *treenetprocnet* R package (Knüsel et al. 2021). TWD quantifies stem shrinkage relative to the previous long-term maximum stem radius (Zweifel et al. 2005). To limit variability due to intraspecific differences in stem shrinkage, we normalized TWD following Peters et al. (2023, 2025). Briefly, for each individual, normalized TWD (TWD_norm_) is calculated by dividing TWD by the maximum daily shrinkage (MDS_max_, μm) during the vegetation period, determined by the 99th percentile of all MDS values over a 5-year period:


(2) \begin{equation*} {TWD}_{norm}=\frac{TWD}{MDS_{max}} \end{equation*}

### Sapflow

Tranzflo compensated heat pulse sap flow sensors were connected in a differential arrangement and utilized to measure time to balance point. This enabled derivation of heat velocity and sap flux. Details of probeset installation, data capture and processing can be found in Quick et al. (2025). Progressively from 2017, in each plot, two oaks were selected, and sap flow sensors were installed on the west- and east- facing sides of the stem at ~1.1 m DBH. Data were collected every 30 min, from 2019, using loggers and multiplexers (CR1000 and AM25T, Campbell Scientific, Logan, UT, USA) in each plot. After data cleaning, only seven sapflowmeters collected constant data between 2019 and 2023.

Sap flux, *VPD* and air temperature 30-min data were used to calculate whole-tree canopy conductance (G_C_). To prevent artefacts, we excluded days with an average daytime *VPD* below 0.3 kPa (Anderegg et al. 2018, Flo et al. 2022) (Anderegg et al. 2018, Flo et al. 2022). We also limited the dataset to daytime hours (09:00 hrs–17:00 h UDT).

Sap flux measurements (L s^−1^) were divided by sapwood area (see Quick et al. 2025) to give sap flux density, F_d_​ (kg s^−1^ m^−2^), and then transformed into molar canopy conductance per unit sapwood area (mol m^−2^ s^−1^). Calculations followed the approach of Monteith and Unsworth (2013) further developed in Phillips and Oren (1998) and Flo et al. (2022):


(3) \begin{equation*} {G}_c=\frac{\left(115.8+0.423{T}_{air}\right){F}_d}{VPD}\bullet \frac{\eta{T}_0}{\left({T}_0+{T}_{air}\right)}\bullet{e}^{-0.00012h} \end{equation*}

where G_C_​ is the canopy conductance (mol m^−2^ s^−1^), T_air_ is top canopy air temperature ( °C), converted to absolute temperature (K) for the temperature correction term in Eq. (3), *VPD* is top canopy vapour pressure deficit (kPa), and *η* is the molar density of air at standard temperature and pressure (44.6 mol m^−3^), used to convert water vapour flux to molar canopy conductance. T_0_​ is the standard temperature, set to 273 K, and *h* the altitude at the site (here: 106 m above sea level, masl). Treatment effects due to eCO_2_ will manifest as either a constant offset in G_C_ or as a departure from slope = −1 in a graph of G_C_ against log(*VPD*).

To compare whether ‘hot events’ impacted G_C_, we plotted G_C_ for 5 days prior the hot event and during the 5 days of the heatwave.

### Terrestrial laser scanning

To assess treatment effects on the vertical distribution of canopy structure, terrestrial laser scanning (TLS) was used to quantify the vertical distribution of projected area across canopy height under ambient and eCO_2_ conditions. Using a RIEGL VZ-400i laser scanner (RIEGL Laser Measurement Systems GmbH, Horn, Austria), three-dimensional point clouds of each experimental patch were collected between January and February 2022. Manual extraction of individual trees from co-registered point clouds were performed in CloudCompare (https://www.cloudcompare.org/). Construction of Quantitative Structure Models (QSMs) and calculation of volumes were performed using the optQSM software (https://github.com/apburt/optqsm) and TreeQSM v.2.4.1 (https://github.com/InverseTampere/TreeQSM). Tree volumes were calculated as cylinders using the sum of bole and branch volume converted into dry mass (Flo et al. 2022, Norby et al. 2024).

### Statistical data analysis

To assess the effects of the hot events on relative growth and short-term legacy effects, linear regression models were fitted over each event period and calculated the slope (growth rate) from the onset of the event through the following month. For example, in 2022, a hot event began on 8 August (mean *T_air_max_*: 28.9  °C; mean *VPD_max_*: 1.4 kPa), and the growth rate ratio was calculated over the period from 8 August to 13 September 2022. Similarly, we extracted growth data of the month prior to each hot events to compare hot events with milder conditions, yet limited bias regarding tree age and disturbance.

All visualizations and analyses were performed in R version 4.4.0 (R Core Team 2017). We used linear mixed-effects models (‘*lmer*’ package in R) to assess the effects of CO_2_ treatment on all our variables (A_net_, g_s_, _i_WUE, g_min_, stomatal density, stomatal length, slope of the growth rate). ‘Treatment’, *T_leaf_* and their interaction were included as fixed effects and ‘Plot’ was included as a random effect in all models with additional fixed or random effects depending on the response variable/dataset: for photosynthetic rate (A_net_) and stomatal conductance (g_s_), ‘Time’ was additionally included as a random effect. In the analysis of minimum leaf conductance (g_min_), ‘Month’ was additionally used as the random effect. Due to the non-normality of data, the relationship between G_C_ and *VPD* and TWD_norm_ was tested using a general linear mixed-effects model with ‘*VPD*’, ‘Treatment’ and ‘Year’ as fixed effects and ‘Plot’ as a random effect. Moreover, TWD_norm_ was square-root transformed to improve model Akaike Information Criterion (AIC). The relationship between G_C_ and *VPD* is non-linear (Grossiord et al. 2017), thus we fitted G_C_ vs *log*(*VPD*). All model assumptions were assessed and validated.

## Results

### Climatic conditions

This study examined five consecutive years, 2019–23, to evaluate the effects of eCO_2_ during a hot-dry event (Figure 1). The year 2022 was characterized by particularly high temperatures (with five consecutive days with air temperatures (T_air_) over 28 °C in August and a maximum T_air_ reaching 35.9 °C in July) and a very low precipitation during summer (2.05 mm from 1 July to 31 August). In contrast, other years experienced comparatively milder temperatures (maximum T_air_ between 31.8 °C in 2020 and 29.1 °C in 2021; Figure 1a) and higher precipitation events (always over 5 mm over the same summer period; Figure 1b), providing a useful baseline for assessing the impact of extreme heat and drought in 2022.

**Figure 1:**
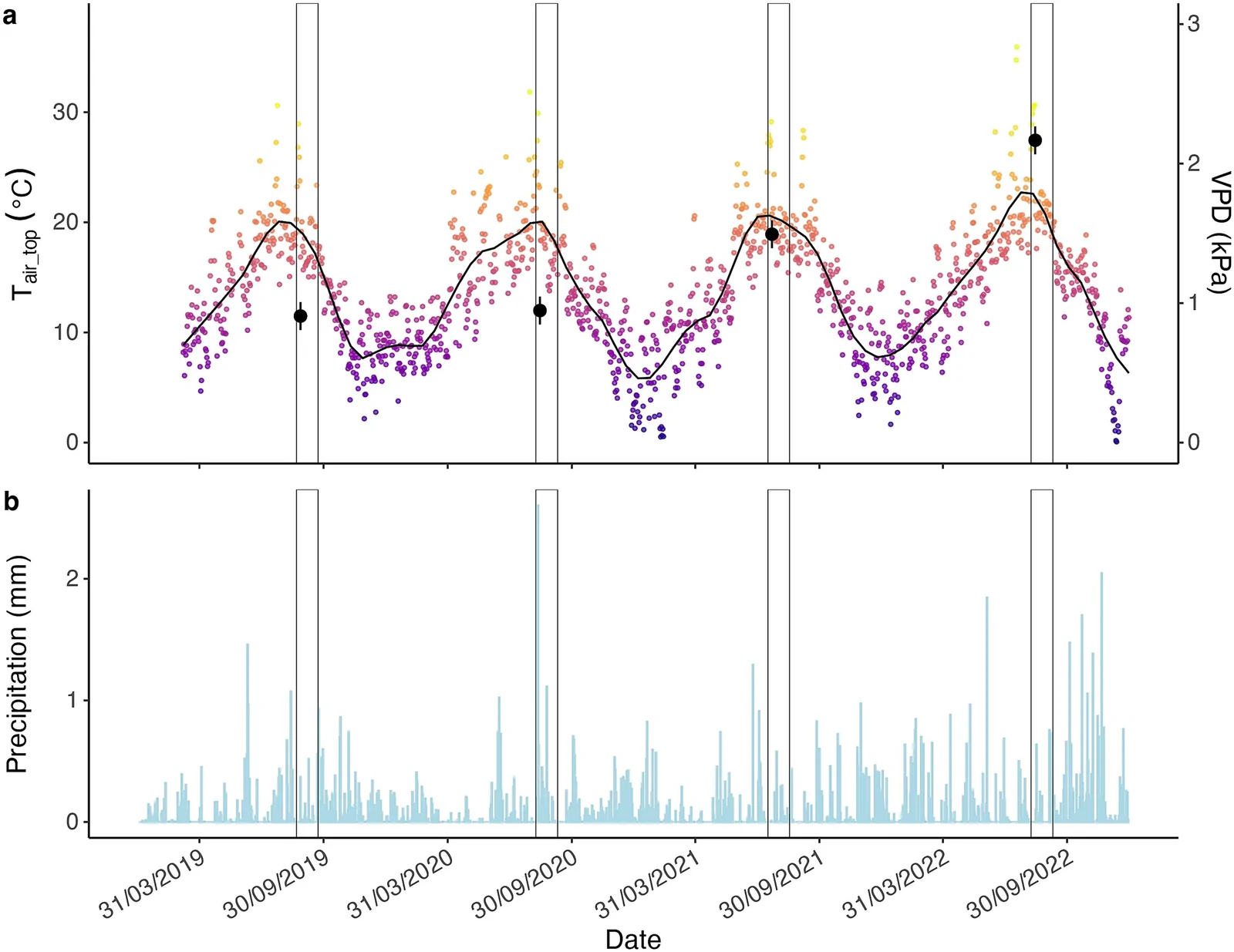
Meteorological conditions at the study site from January 2019 to January 2023. (a) Daily mean canopy-top air temperature ( °C), colour coded from purple (cool) to yellow (warm), with a black smoothed trend line indicating the 5-day running mean. Large black points represent 5-day mean *VPD* ± SD. (b) Daily precipitation (mm) over the same period. Grey boxes indicate defined ‘hot events’ (>5 consecutive days of near-maximum annual temperature) and 1 month following each event.

### Leaf level physiology

Trees grown under eCO_2_ exhibited significantly higher net photosynthetic assimilation (A_net_) compared with those under aCO_2_ (+26%) (*P* < 0.01; Table 2, Figure 2), indicating that the enhanced photosynthetic performance previously observed during the early years of BIFoR FACE remained evident following ~8 years of CO_2_ enrichment. For all years stomatal conductance (g_s_) showed a tendency to be lower in eCO_2_ trees than under aCO_2_. Intrinsic water-use efficiency (_i_WUE = A_net_/g_s_) was significantly higher (+9%) (*P* < 0.05) in eCO_2_. Moreover, iWUE significantly increased with leaf temperature (T_leaf_) in aCO_2_ trees whilst such sensitivity was not detected in eCO_2_ trees (Figure 2b). This increase was mainly due to a decrease in g_s_ with T_leaf_ in aCO_2_.

**Table 2:** Mean values (± standard error) of photosynthetic and stomatal traits measured under ambient (aCO_2_) and elevated (eCO_2_) atmospheric CO_2_ conditions. Parameters include net photosynthetic rate (A_net_), stomatal conductance (g_s_), minimum leaf conductance (g_min_), stomatal density and stomatal length. *P*-values indicate the results of statistical comparisons between treatments. Units are as follows: A_net_ (μmol m^−2^ s^−1^), g_s_ (mol m^−2^ s^−1^), _i_WUE (μmol CO_2_ mol^−1^ H_2_O), g_min_ (mmol m^−2^ s^−1^), stomatal density (stomata mm^−2^) and stomatal length (μm).

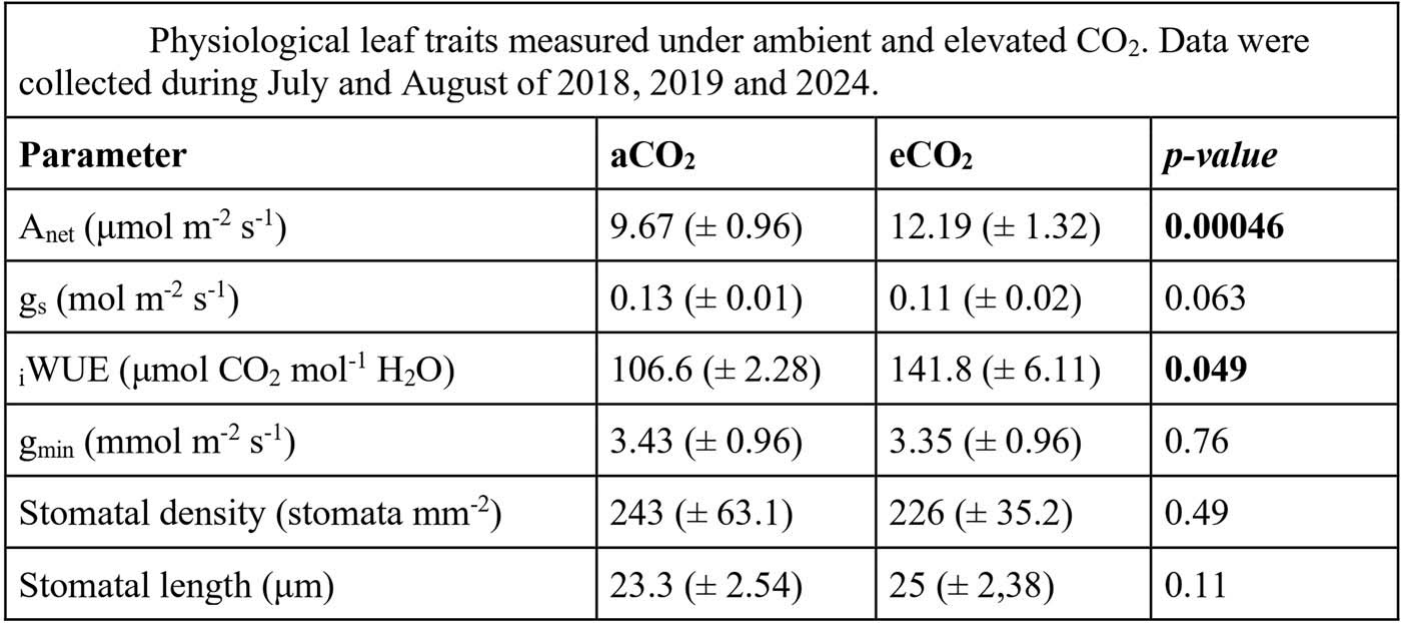

Bold values indicate statistically significant differences between CO_2_ treatments (*P* < 0.05).

**Figure 2:**
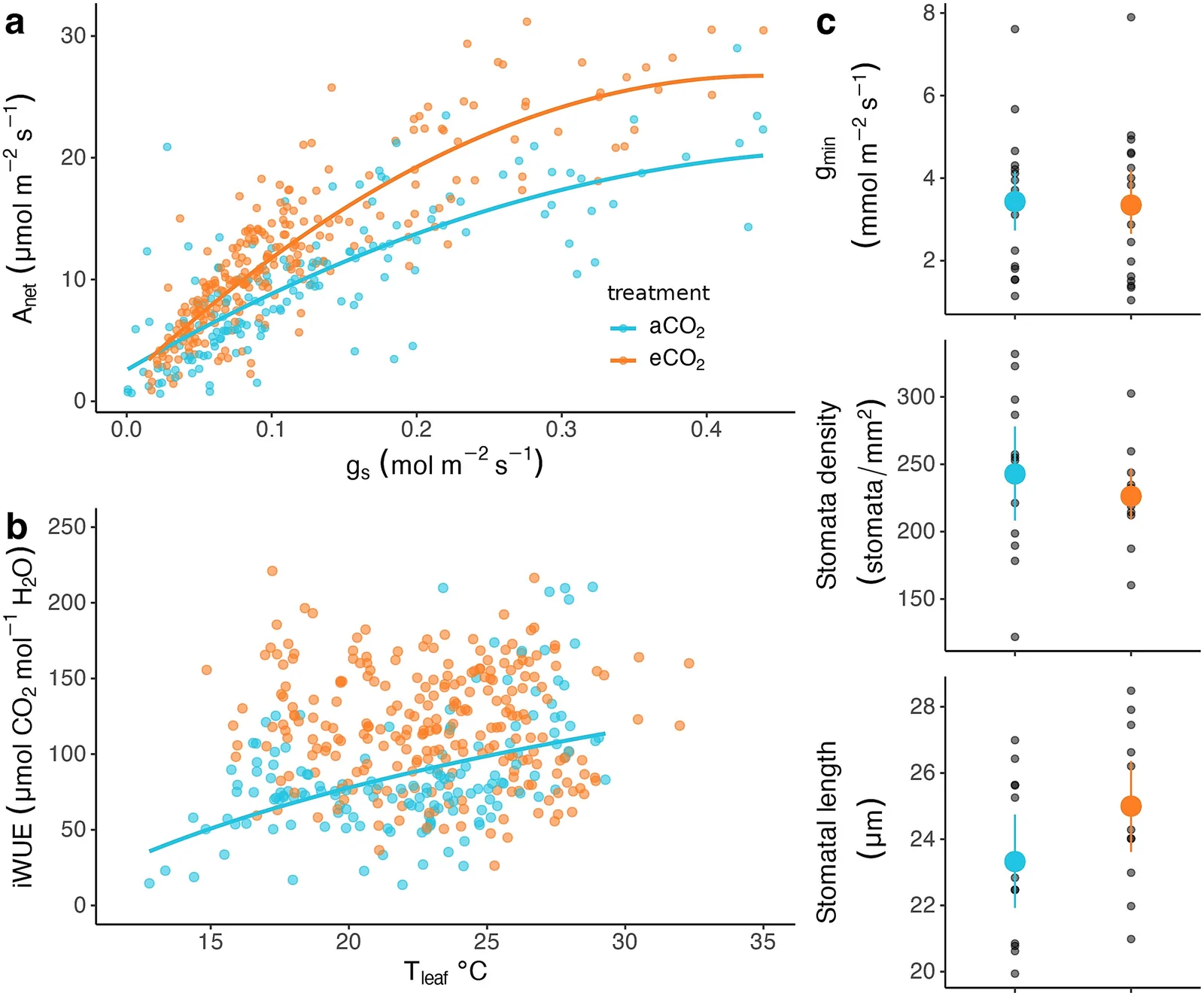
(a) Relationship between net photosynthetic assimilation (A_net_) and stomatal conductance (g_s_) and (b) relationship between intrinsic water-use efficiency (_i_WUE) and leaf temperature (T_leaf_) in mature *Quercus robur* in either ambient CO_2_ (aCO_2_) (blue) or elevated CO_2_ (eCO_2_) (orange) conditions. Data were collected during July and August of 2018, 2019 and 2024. Only significant relationships are plotted. (c) Minimum stomatal conductance (g_min_), stomatal density and stomatal length. Each data point represents diurnal spot measurements collected from three aCO_2_ plots and three eCO_2_ plots. Solid coloured lines indicate CO_2_ treatment, with blue representing aCO_2_ and orange representing eCO_2_.

No significant differences were observed between CO_2_ treatments for minimum stomatal conductance (g_min_; *P* = 0.76), stomatal density (*P* = 0.46) or stomatal length (*P* = 0.30; Table 2, Figure 2c).

### Tree growth

The slopes of stem growth rate ratios (*m_growth*) during the short-term recovery post hot events did not differ significantly between eCO_2_ and aCO_2_ treatments in 2019, 2020 and 2021 (Figure 3; all *P* > 0.05). Nevertheless, *m_*growth in aCO_2_ trees was consistently lower than in eCO_2_ trees, suggesting a subtle, persistent trend of increased growth under eCO_2_ following the highest summer temperatures. During the most severe heat event in 2022, *m*_growth was significantly lower in aCO_2_ trees (*P* = 0.0308, Figure 3), indicating a markedly reduced growth rate under aCO_2_ following extreme conditions.

**Figure 3:**
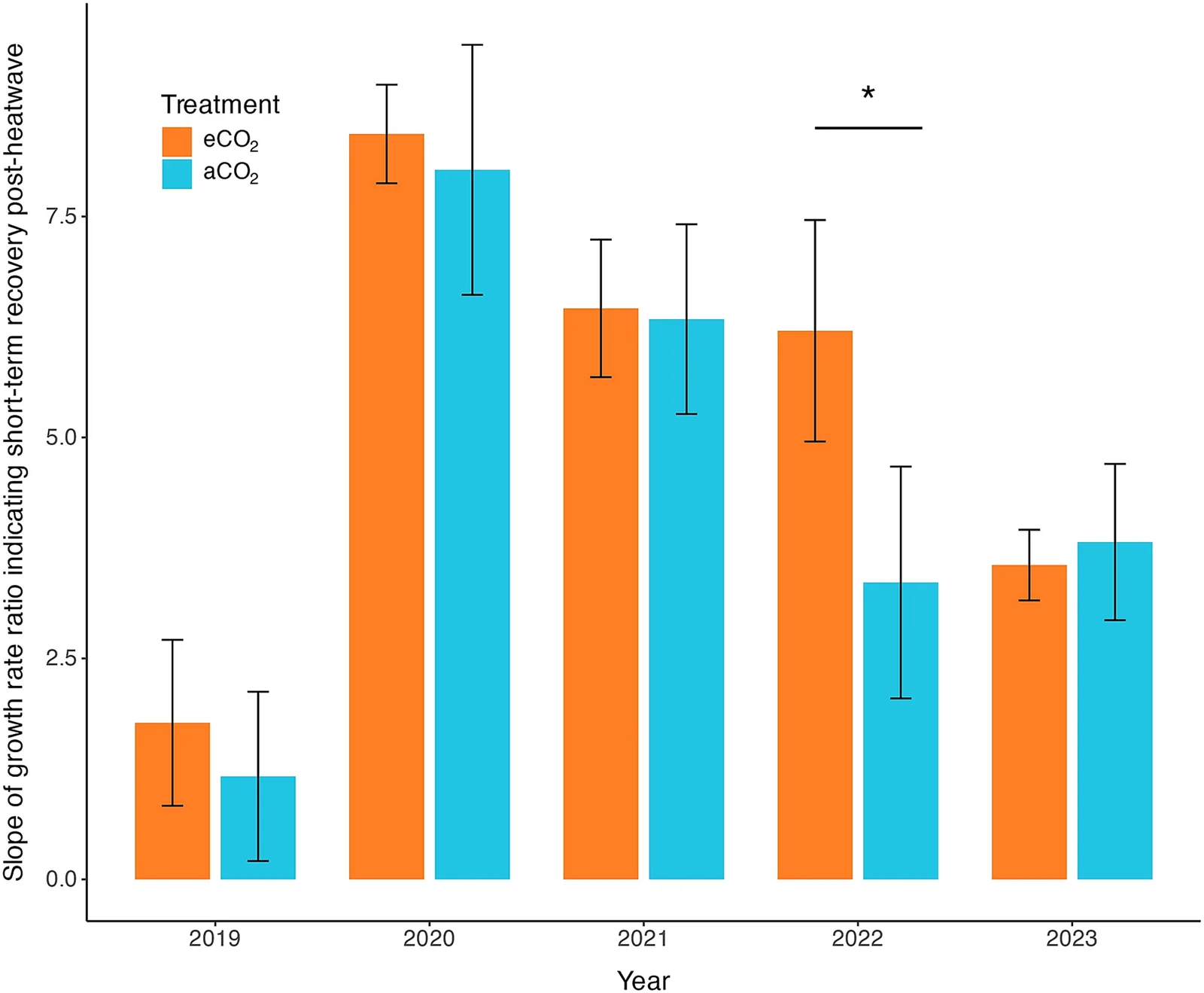
Stem growth ratio (*m*_growth) during hot events from 2019 to 2023 under ambient CO_2_ (aCO_2_, blue) and elevated CO_2_ (eCO_2_, orange) treatments. Hot events are defined as 30-day periods preceded by five consecutive days with maximum daily temperatures (see Materials and methods). Stem growth ratio reflects relative stem growth following these events, allowing comparison between CO_2_ treatments across years. Bars represent mean values, and error bars indicate ± standard error (SE). Significant differences between treatments at *P* < 0.05 are indicated by an asterisk.

Prior to any hot events, *m*_growth was not significantly different between aCO_2_ and eCO_2_ treatment for any years (see Figure S2 available as Supplementary Data at *Tree Physiology* Online) indicating that the reduction observed was likely due to the intensity of the hot events, especially 2022.

Across all years, no significant year-to-year differences of *m_growth* were detected within aCO_2_ and eCO_2_ treatments, except for 2019 (Figure 3). This lower *m*_growth was likely due to a severe herbivory event from winter moth (*Operophtera brumata*) infestation that occurred at the site in 2018. This herbivory disturbance may have impacted carbon uptake and decreased reserves used for next year (i.e., 2019) growth.

### Canopy conductance

Across all years, G_C_ declined significantly with increasing *VPD* (all *P* < 0.05; Figure 4). Overall, no significant main effect of CO_2_ treatment was observed on G_C_ (*P* = 0.92). When averaging across treatments, G_C_ was slightly lower during heat events compared with values measured directly prior each heat events (−2.5%; *P* = 0.25).

**Figure 4:**
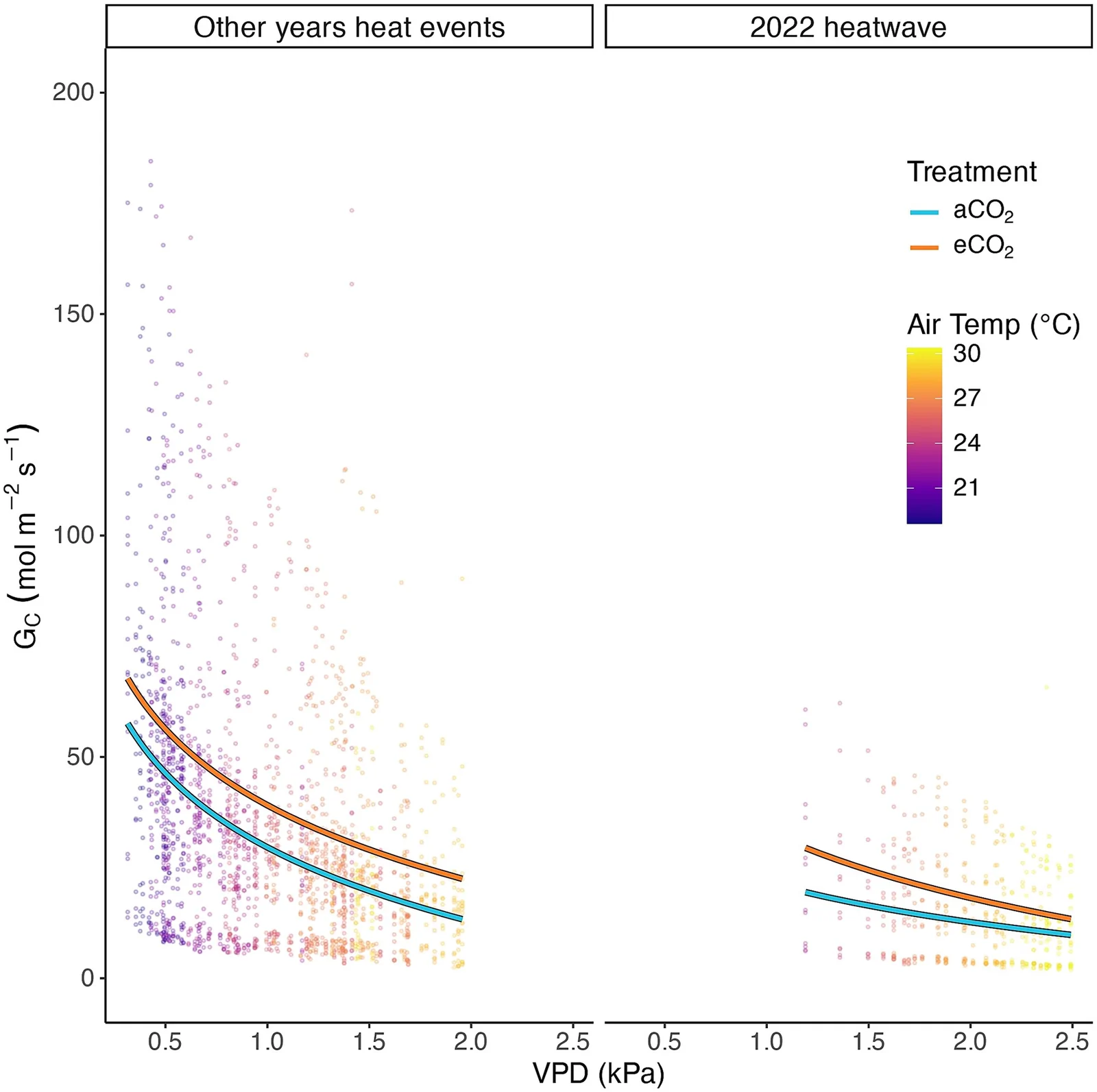
Relationship between canopy conductance (G_C_, mol m^−2^ s^−1^) and vapour pressure deficit (*VPD*, kPa) in mature *Quercus robur*, based on sap measurements. Facets represent a separate time periods: ‘Other years heat events’ (2019–21 and 2023) and ‘2022 heatwave’ (2022). Data points represent half-hourly mean measurements between 09:00  and 17:00 h local time 00 h UDT (see Materials and methods), for ambient CO_2_ (aCO_2_, blue, *n* = 5) and elevated CO_2_ (eCO_2_, orange, *n* = 3) trees. Lines represent fitted relationshipspredicted by the *glmer* model. Slopes (*m_cond*) were more negative under eCO_2_ compared with aCO_2_, indicating greater overall sensitivity of Gc to increasing *VPD* under eCO_2_.

G_C_ sensitivity to *VPD* (*m_cond;* i.e., slope of relationship between log(G_C_) and *VPD*) was significantly lower during heat events (*P* < 0.001). Although there was no significant differences between treatments (*P* = 0.71), *m_cond* showed a consistent lower trend in eCO_2_ (average −25.6 across all years) compared with aCO_2_ treatment (average − 19.4 across all years), suggesting greater overall G_C_ sensitivity to *VPD* under eCO_2_. However, differences between treatments were more pronounced at higher *VPD* levels prior to heat events, indicating that treatment effects were not uniform across the full *VPD* range. In 2022, *m_cond* became less negative compared with previous heat events, suggesting that sensitivity was reduced, probably due to earlier stomatal closure and lower overall G_C_ during the extreme heatwave. Whilst not significant, *m_cond* decreased by 50% in aCO_2_ treatment, but only by 27% in eCO_2_ treatment between previous years’ ‘hot events’ and the 2022 heatwave (Figure 4), suggesting that eCO_2_ trees maintained comparatively greater stomatal sensitivity to *VPD* under extreme conditions.

### Tree water deficit

In both treatments, TWD_norm_ increased with *VPD* and T_air_ (*P* < 0.01; Figure 5a). During the hot events of 2019–2021 and 2023, the slopes of TWD_norm_ vs *VPD* were similar between aCO_2_ and eCO_2_ (*P* = 0.37). However, during the heatwave of 2022 eCO_2_-grown treatment showed a numerically steeper increase in TWD_norm_ with *VPD* compared with aCO_2_ treatment (0.601 in eCO_2_ vs 0.537 in aCO_2_)(Figure 5a) although this difference was not statistically significant (*P* = 0.181). Overall, TWD_norm_ was significantly lower in eCO_2_ treatment between July and October across all years (*P* = 0.039; Figure 5b), yet during the 2022 heatwave, TWD_norm_ was significantly higher than other years (*P* < 0.001) and although there was no difference between treatments, the peak of TWD_norm_ reached higher values in eCO_2_ treatment.

**Figure 5:**
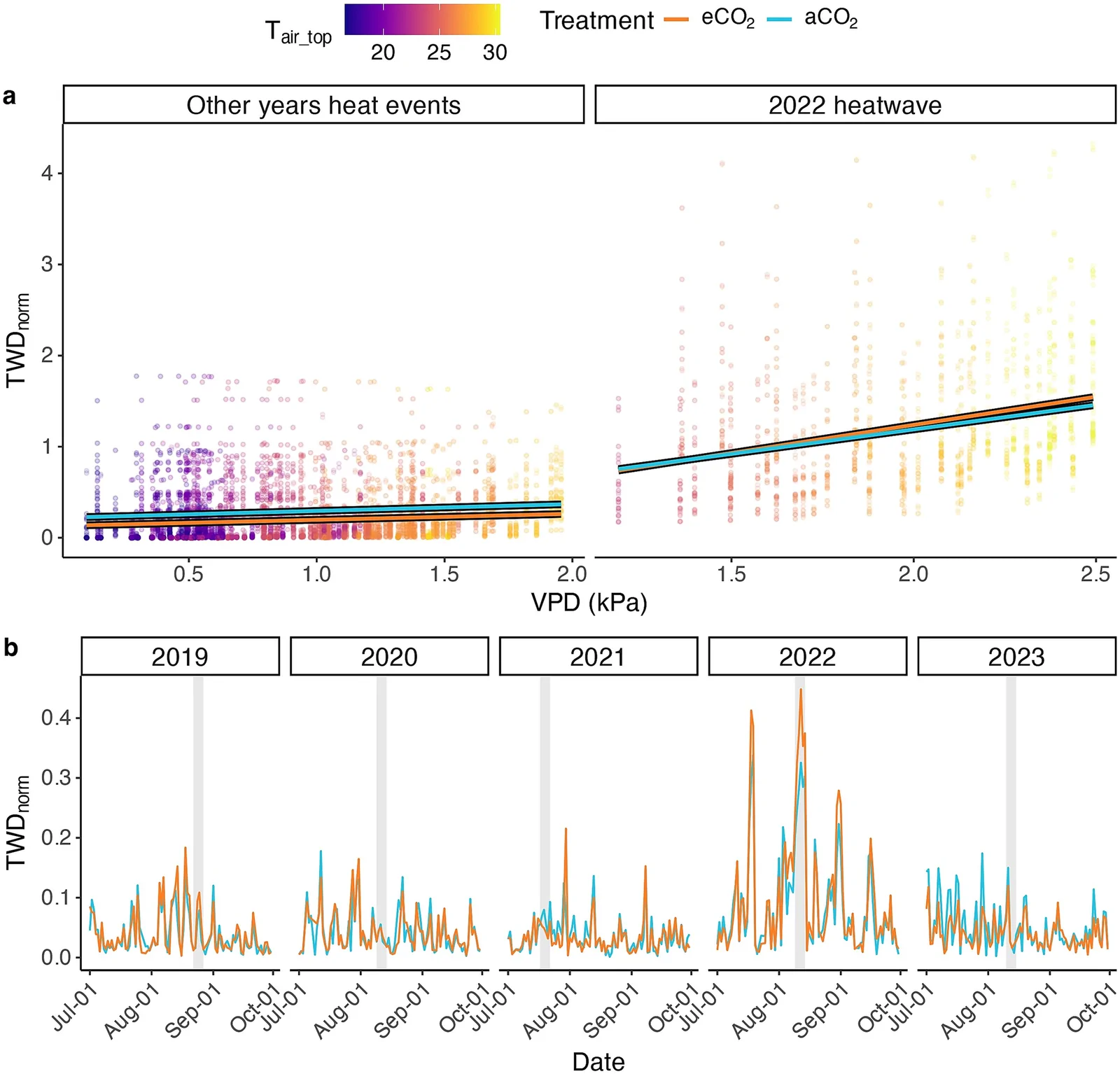
(a) Relationship between normalized tree water deficit (TWD_norm_) and *VPD* (kPa). Each point represents an hourly observation for periods prior to heat events, coloured as a gradient of air temperature (T_air_), with purple indicating coolest and yellow as the warmest temperatures. Facets represent a separate time periods: ‘Other years heat events’ (2019–21 and 2023) and ‘2022 heatwave’ (2022). Lines represent fitted relationships across all data points for aCO_2_ (blue) and eCO_2_ (orange) trees. During hot events in 2019–21 and 2023, slopes were similar between treatments (*P* = 0.37). During the 2022 heatwave, TWD_norm_ increased more steeply with *VPD* in eCO_2_ trees compared with aCO_2_ trees although this was not statistically significant (*P* = 0.181). (b) Seasonal dynamics of normalized TWD (TWD_norm_) in summer periods from 2019 to 2023 averaged per treatment. Lines represent daily treatment means over time with blue indicating ambient CO_2_ (aCO_2_) and orange indicating elevated CO_2_ (eCO_2_). Overall, TWD_norm_ was significantly lower in eCO_2_ trees between July and October across all years (*P* = 0.039).

## Discussion

### Elevated CO_2_ partially buffers short-term heat-induced growth reductions

In our study, trees exposed to eCO_2_ consistently exhibited higher carbon assimilation rates compared with those under aCO_2_ conditions in 2018, 2019 and 2024. Previous research at the BIFoR FACE site has shown that this enhanced assimilation translates into ~10% greater woody biomass production under eCO_2_ conditions (Norby et al. 2024, Norby 2025). These findings are consistent with broader evidence reporting that eCO_2_ can stimulate tree growth, although the magnitude of response can vary across species and forest types. We found that, in 2022, an extreme heat event reduced tree growth in both CO_2_ treatments (>20%), but the decline was more pronounced under aCO_2_. Following extreme heat, *Q. robur* under eCO_2_ recovered more rapidly, exhibiting significantly higher growth rates (Figure 3). Previous studies have reported similar responses in seedlings and controlled environments, where heat stress reduces photosynthesis and growth, with these reductions less pronounced under eCO_2_ (Ameye et al. 2012, Bauweraerts et al. 2013). Importantly, our results demonstrate that eCO_2_ can partially mitigate growth reductions following extreme heat events in mature trees under natural field conditions, addressing a key gap in current understanding. This buffering effect may reflect enhanced carbon availability under eCO_2_, allowing trees to recover growth rapidly. Increased stomatal sensitivity to *VPD* in eCO_2_ may also help prevent hydraulic damages under high atmospheric demand, although this may increase reliance on internal water reserves, as reflected by higher TWD.

Whilst annual growth is partly influenced by carbon assimilation from the previous year, the reduction of growth during 2022 was likely driven by acute water and heat stress, as reflected by higher TWD and extreme T_air_ during the event (Figure 5b). Although carbon allocation to stem growth can lag behind assimilation, wood biomass increment also responds strongly to current-year climate variability (Teets et al. 2022). In our study, TWD_norm_ stayed relatively low across all years (TWD_max_ < 500 μm) except in 2022 when peak TWD_norm_ exceeded 1000 μm, coinciding with a complete halt in growth (see Figure S3 available as Supplementary Data at *Tree Physiology* Online). Together, these findings indicate that the 2022 heatwave imposed strong hydraulic constraints on growth. However, the 2022 heat stress did not appear to have a lasting effect on tree growth in 2023, with trees in both treatments reaching their highest end-of-year growth (see Figure S4 available as Supplementary Data at *Tree Physiology* Online). This suggests that, although growth was temporarily suppressed, trees retained the capacity to recover following the heat event.

### Increased photosynthesis is the principal driver of CO_2_-enhanced intrinsic water-use efficiency

Our findings indicate that trees grown under eCO_2_ exhibited enhanced _i_WUE compared with those grown under aCO_2_ in all studied years (2018, 2019 and 2024; Figure 2a, Table 2). At our site, this increase in _i_WUE was primarily driven by increases in A_net_. This pattern is consistent with previous studies showing that increases in _i_WUE under eCO_2_ are often driven by enhanced photosynthesis rather than reductions in g_s_ (Guerrieri et al. 2019). Enhanced _i_WUE suggests that eCO_2_-grown trees may slightly reduce overall water consumption, whilst increasing carbon uptake, thereby maintaining carbon assimilation under both non-stressful and stressful conditions when compared with aCO_2_ grown trees. These results are consistent with previous FACE-based studies reporting increases in _i_WUE in multiple tree species (Battipaglia et al. 2013, Gardner et al. 2022) as well as broader syntheses of eCO_2_ experiments of trees by Wang and Wang (2021).

In this study, no changes in stomatal density, size or minimum stomatal conductance (g_min_) were observed in *Q. robur* (Figure 2b), suggesting that the increase in _i_WUE under eCO_2_ was primarily driven by physiological changes (e.g., increases in A_net_ and slight reductions in g_s_), rather than morphological adjustments. These physiological responses provide important context for interpreting the canopy-scale responses observed during heat events.

### Elevated CO_2_ alters canopy conductance sensitivity to *VPD* and TWD_norm_ responses during extreme heat events

Increased _i_WUE under eCO_2_ is typically expected to translate to a reduction in water consumption if iWUE is driven by reductions in g_s_ and therefore leads to reduced canopy conductance (G_C_) (Zhan et al. 2025). However, we observed no significant decrease in G_C_ with eCO_2_. This may reflect increased leaf biomass offsetting reductions in leaf-level g_s_, resulting in similar whole-tree water use (Curtis and Wang 1998).

Despite the lack of response of G_C_ to eCO_2_, our results indicate that G_C_ was consistently more sensitive to increased *VPD* in eCO_2_-grown trees (more negative *m_cond* values) (Figure 4). This increased sensitivity was also evident during heat events. This suggests that CO_2_-enhanced _i_WUE may increase stomatal sensitivity to elevated *VPD* and temperature, potentially closing stomata at a faster rate than aCO_2_ trees. These findings agree with previous studies that show increased sensitivity to *VPD* under warming in a wide range of species (e.g., in temperate deciduous Heath 1998; in semi-arid evergreen Grossiord et al. 2017; in coffee Avila et al. 2020), and, to our knowledge, this is the first study to show that eCO_2_ could decrease this effect in *Q. robur*. This strategy may allow water savings at high *VPD* at the cost of slightly reduced A_net_. Such mechanisms have important implications for water and carbon dynamics in a warmer world as CO_2_-driven gains in _i_WUE may diminish with increased *VPD* (Chai et al. 2024).

During the daytime, trees lose water via transpiration, and when this loss exceeds soil water supply, internal water reserves are mobilized, leading to TWD (Knipfer et al. 2019, Zweifel et al. 2021, Ziegler et al. 2024). This mechanism also occurs with increased *VPD* (Oberhuber et al. 2023), often halting growth and inducing TWD. In our study, we observed a sharp increase in TWD_norm_ with *VPD*, which was more noticeable under eCO_2_ compared with aCO_2_ (see Figure 5), possibly due to increased G_C_ sensitivity. Under extreme heat conditions (i.e., 2022) increased evaporative demand likely accelerated tissue dehydration, even where stomatal responses were strongly regulated. Closing stomata aperture at increased *VPD* may therefore increase reliance on internal water reserves. Such responses may become increasingly important under hotter and drier conditions, as accumulated TWD can increase the risk of hydraulic failure if night-time rehydration is limited (Andriantelomanana et al. 2024).

### Elevated CO_2_ moderates heat stress, but limitations remain

Our findings demonstrate the capacity of eCO_2_ to mitigate some of the negative impacts of climate extremes, particularly heatwaves, on the productivity of long-established temperate forest. However, these benefits are not universal and may diminish under concurrent stressors such as drought or high *VPD*. Importantly, much of the existing experimental evidence derives from short-term studies on seedlings conducted under controlled conditions, which may not fully represent the responses of mature trees in natural ecosystems and long-term field experiments. Our ecosystem-scale findings using FACE clarify interactions between CO_2_, temperatures and *VPD* in mature forests that are difficult to capture in more controlled experiments. Whilst some FACE experiments (e.g., EucFACE) have shown limited aboveground biomass responses, likely due to phosphorus limitation (Crous et al. 2015), the BIFoR FACE site has not exhibited clear nutrient constraints (Gardner et al. 2022). Moreover, at BIFoR FACE, increased carbon assimilation under eCO_2_ has resulted in greater aboveground biomass production (Norby et al. 2024), although woody structural responses were less clear, suggesting that additional carbon may be allocated to non-structural or belowground pools rather than canopy expansion. Stand age and forest structure are also likely to influence responses to eCO_2_. BIFoR FACE comprises mature, long-established, mixed deciduous trees, in contrast to earlier FACE experiments (e.g., ORNL, DUKE) conducted on younger, even-aged plantations, which may respond more uniformly to eCO_2_. Taken together, these observations reinforce the need for long-term, ecosystem-scale studies to constrain forest responses to rising atmospheric CO_2_ and improve predictions of the global carbon cycle.

## Conclusion

Our findings demonstrate that long-term exposure to eCO_2_ has measurable effects on tree physiological function, water regulation and growth, influencing responses to increased temperatures. Over 8 years, eCO_2_ consistently enhanced _i_WUE and supported greater tree woody biomass production. However, rather than uniformly improving performance, eCO_2_ altered the balance between carbon gain and water use in context-dependent ways, supporting growth under ambient conditions whilst providing only partial buffering during periods of climatic stress.

Despite this, hot-dry events still reduced annual growth (Figure 3, Figure S4 available as Supplementary Data at *Tree Physiology* Online), highlighting the limitations of eCO_2_-induced benefits under extreme climatic stress. Furthermore, during hot-dry events, trees exposed to eCO_2_ exhibited altered sensitivity to *VPD* and TWD (Figures 4 and 5), indicating shifts in stomatal regulation and water use strategy relative to those under aCO_2_.

Importantly, our results suggest that eCO_2_ may partially mitigate the negative impacts of increasing temperatures on forest productivity. Our findings emphasize the importance of accounting for interactions between atmospheric CO_2_ and climate extremes, and provide testable constraints on model forecasts of forest responses to climate change. Incorporating these dynamic responses into ecosystem models will be critical for improving predictions of water fluxes, carbon cycling and long-term ecosystem resilience.

## Supplementary Material

Supplementary_Gardner_et_al_2026_Tree_Physiology_Revision_tpag114

## Data Availability

The data underlying this article will be shared on reasonable request to the corresponding authors.
