# Supplementary material for: Elevated CO2 buffers negative impacts of heat stress on short-term growth adjustments of 180-year-old Quercus robur
Source: Tree Physiol. 2026 Aug 20;46(10):tpag114. doi: 10.1093/treephys/tpag114 (PMC13669954; doi:10.1093/treephys/tpag114)
Supplement: Supplementary file 1 [file supplementary_gardner_et_al_2026_tree_physiology_revision_tpag114.docx]

**Supplementary Information for Gardner et al. Tree Physiology**

**Fig S1.** Schematic diagram showing the timeline of data collection and abiotic and biotic stress events that occurred at BIFoR FACE between 2018 and 2024.

**Fig. S2.** Stem growth ratio (*m*_growth) pre heat event window from 2019 to 2022 under ambient CO₂ (aCO_2_, blue) and elevated CO_2_ (eCO_2_, orange) treatments. Hot events are defined as 30-day periods preceded by five consecutive days with maximum daily temperatures (see methods). Stem growth ratio reflects relative stem growth during and following these events, allowing comparison between CO_2_ treatments across years.

**Fig. S3**. Tree water deficit (TWD) (left axis; points) and growth (right axis; lines) for eCO_2_ and aCO_2_ trees between June and September of 2019, 2020, 2021 and 2022.

**Fig. S4.** Seasonal stem growth accumulation (µm) of mature *Quercus robur* trees across day of year (DOY) under ambient CO₂ (aCO₂; blue) and elevated CO₂ (eCO₂; orange) conditions between 2019 and 2023 at the BIFoR FACE facility. Panels represent individual study years. The secondary y-axis indicates relative growth progression across the growing season. Lines represent treatment-level mean growth trajectories for each year.
